# Letter from Dr. J. Murray

**Published:** 1852-10

**Authors:** J. Murray

**Affiliations:** Bridgewater, Pa.


### Correspondence.

Dr. Taylor,—Dear Sir :—From the great difficulty attending the insertion of whole upper sets of teeth, on atmospheric pressure or suction, my attention has been turned lately to that subject. I have been making some experiments, which I think have resulted in throwing some light upon the subject, that may prove useful to the profession.

The general practice, so far as I have had opportunity of making observations, is, to cover the whole roof of the mouth, or nearly so, with the plate. This is objectionable.

1st. From the fact, that the greater the surface to be covered the more difficult it is to make a perfect fit.

2. Because the plate covers so much of the palate, that one of the principle pleasures arising from the comminution of our food, is greatly reduced, or almost destroyed.

3. Because it is an unnecessary weight in the mouth. And,

4. Because a smaller plate will answer *better*.

I have proved to my satisfaction, by the experiments alluded to, that a plate covering a surface as large as an English shilling, is sufficient to retain an entire upper set of teeth in the mouth. Let an air-chamber the size of a half-dime, be made in the center of the plate, with its edges well fitted to the palate, and it is not particular about the rest of the plate being air-tight; though for comfort and perfection, it should be accurately fitted.

Now my plan is simply this, I make a plate of the above named size, accurately fitted, with an air-chamber. If I desire to mount scattered teeth, I do it by arms extending from the edge of the plate to the place, or places in the mouth wanting teeth. By this means I dispense with clasps altogether. A single tooth can be inserted in this way, without the use of clasps. If I desire to mount two or three together, I extend the plate over the gum. But if a whole set is required on the upper jaw, I swedge a mere cap for the gum, making it fit so well that it will be air-tight itself, and then by three arms I attach it to the air-tight plate, which will hold it with perfect security. The firmness of the plate, does not depend upon the accuracy of the fit on the labial side of the gum, as it is thought, by Dr. Harris and others, but upon the air-chamber itself.

I claim that this method posesses the following advantages,

1. It requires less gold.
2. It is scarcely perceptible in the mouth.
3. Clasps, that are so destructive to the teeth upon which they are fastened, are altogether dispensed with.
4. And last, but not least, it permanently secures the teeth in the mouth.

I will merely add, I have a plan in my mind, if successful, that will enable us to dispense with the plate, in the palate, making the cap upon the gum answer every purpose. J. MURRAY,

*Bridgewater, Pa.*

